# Anti-Thymocyte Globulin Prophylaxis in Patients With Hematological Malignancies Undergoing Allogeneic Hematopoietic Stem Cell Transplantation: An Updated Meta-Analysis

**DOI:** 10.3389/fonc.2021.717678

**Published:** 2021-08-20

**Authors:** Xue Yang, Dongjun Li, Yao Xie

**Affiliations:** ^1^Department of Pediatrics, West China Second University Hospital, Sichuan University, Chengdu, China; ^2^Key Laboratory of Birth Defects and Related Diseases of Women and Children, Sichuan University, Ministry of Education, Chengdu, China; ^3^Department of Obstetrics and Gynaecology, Sichuan Provincial People’s Hospital, University of Electronic Science and Technology of China, Chengdu, China; ^4^Chinese Academy of Sciences, Sichuan Translational Medicine Research Hospital, Chengdu, China

**Keywords:** anti-thymocyte globulin, hematological malignancies, allogeneic hematopoietic stem cell transplantation, overall survival, meta-analysis

## Abstract

**Background:**

Anti-thymocyte globulin (ATG) prophylaxis reduces graft-*versus*-host disease (GVHD) incidence. This meta-analysis aimed to explore the long-term efficacy of ATG and the influencing factors in patients undergoing allogeneic hematopoietic stem cell transplantation (allo-HSCT).

**Methods:**

PubMed, Embase, and Cochrane databases were searched for the relevant studies published up to August 2020. Data from randomized controlled trials (RCTs) on ATG prophylaxis for GVHD prevention in allo-HSCT patients were extracted.

**Results:**

A total of eight relevant RCTs (1,348 patients) were included. ATG significantly reduced the incidence of grade III–IV aGVHD (P = 0.001) and cGVHD (P < 0.001). ATG significantly improved the GVHD relapse-free survival (GRFS) (P < 0.001). The immunosuppressive regimen (number and dose of immunosuppressants) was significantly reduced when using ATG (P = 0.005). Epstein-Barr virus (EBV) reactivation was high in patients receiving ATG (P = 0.003). No significant differences were detected in relapses, overall survival (OS), relapse-free survival (RFS), and non-relapse mortality (NRM) between the ATG and no ATG groups. Subgroup analyses revealed that the donor type and ATG formulation might be the possible sources of heterogeneity among the included studies. Meta-regression analysis showed that the cumulative dose of ATG did not affect GVHD, OS, relapse, RFS, and NRM.

**Conclusion:**

Although ATG had no significant effect on relapse, RFS, and NRM, it significantly reduced the occurrence and severity of GVHD, improved the GRFS, and reduced the number and dose of immunosuppressants in patients undergoing allo-HSCT.

## Introduction

Hematopoietic stem cell transplantation (HSCTs) is a major treatment option for patients with malignant blood diseases, but the clinical management of post-transplantation complications can be challenging (1, 2). Graft-*versus*-host disease (GVHD) is the major life-threatening complication following allo-HSCT (3, 4). Drug-related toxicity; infections by viruses bacteria, and fungi; immune-mediated disorders; and disease recurrence can also lead to potentially lethal complications (5, 6). Still, the acute GVHD (aGvHD) rates have significantly improved in recent years (7).

Different anti-thymocyte globulin (ATG) formulations can be used for T-cell depletion, thereby reducing the risk of rejection in allo-HSCTs (8). The addition of ATG to standard cyclosporine A and methotrexate prophylaxis is widely applied for GVHD prevention prior to allo-HSCT (9). Still, the long-term efficacy of ATG for allo-HSCT is yet to be elucidated. Several studies evaluated the efficacy of ATG plus standard GVHD prophylaxis in patients with hematological malignancies who received myeloablative conditioning (MAC) or reduced-intensity conditioning (RIC) regimen from related and unrelated donors prior to allo-HSCT (10–12). A meta-analysis suggested that ATG significantly reduces the risk of acute and chronic GVHD, whereas the impact of ATG on disease relapse and the survival of patients was unsure (13). The effect of ATG on long-term prognosis has not been analyzed in previous meta-analyses (8, 13–15). The composite endpoints of GVHD relapse-free survival (GRFS) and free of immunosuppressive therapy (i.e., immunosuppressants are no longer necessary) are considered indicators of allo-HSCT completion (10, 16, 17). In addition, there was a lack of in-depth analysis to identify factors that affect the efficacy of ATG. Recently, three additional randomized controlled trials (RCTs) assessing long-term outcomes have been published. Walker et al. showed that the addition of ATG resulted in improved 2-year survival and decreased use of immunosuppressive therapy after unrelated donor transplantation (16). Chang et al. found an improved 3-year GRFS in patients undergoing sibling donor transplantation (10). Bonifazi et al. reported that patients receiving sibling donor transplantation showed improved 5-year GRFS and short immunosuppressive treatment, whereas the overall survival (OS) did not differ significantly (17).

This updated meta-analysis aimed to assess the impact of ATG therapy on acute and chronic GVHD, OS, GRFS, free of immunosuppressant therapy, relapse, RFS, non-relapse mortality (NRM), and infections, the effect of the optimal donor source, and the formulation of ATG in allo-HSCT.

## Materials and Methods

### Literature Search Strategy

This meta-analysis was carried out according to the Preferred Reporting Items for Systematic Reviews and Meta-Analyses (PRISMA) guidelines (18). A systematic search was performed from databases including PubMed, Embase, the Cochrane Library, and Clinical Trials Registry for potentially eligible studies published up to August 2020. The key terms “hematopoietic stem cell,” “allogeneic hematopoietic cell transplantation,” and “anti-thymocyte globulin” were used to identify the relevant publications. The reference lists from the retrieved studies were reviewed to identify any additional eligible studies.

### Study Selection

The inclusion criteria were as follows: (1) population: stem cell transplantation for a hematological malignancy; there were no restrictions on matching during this screening process as long as the patients were undergoing allo-HSCT; (2) intervention: ATG and standard GVHD prophylaxis; (3) control: standard GVHD prophylaxis; (4) outcomes: III–IV aGVHD, cGVHD, OS, GRFS, free immunosuppressant therapy (no longer needing immunosuppressant therapy), the incidence of relapse, RFS, NRM, and infections; (5) study design: RCTs; and (6) language was limited to English. All steps of the study were conducted independently by two investigators (XY and DL).

### Data Extraction and Quality Assessment

The study characteristics, including the first author’s name, NCT register ID, sample size, age and sex of the patients, donor source, intervention arms, ATG formulation and dose, follow-up, and outcomes, were extracted by two authors (XY and DL) independently. If a study reported hazard ratio (HR), the data of HR and 95% confidence interval (CI) were extracted. Otherwise, the risk ratio (RR) and 95% CI with events in two arms were calculated. The level of evidence of all articles was assessed independently by two authors (XY and DL) according to the Cochrane Handbook. Any discrepancy was resolved by discussion.

### Statistical Analysis

RRs and corresponding 95% CIs were used to summarize the data. Statistical heterogeneity among studies was calculated using Cochran’s Q test and the I^2^ index (19). Since the population originated from different countries and there was heterogeneity in the interventions, the random-effects model was used. Subgroup analyses according to donor source (related *vs.* unrelated donors) and ATG formulation (ATG thymoglobulin *vs.* anti-T lymphocyte globulin (ATLG)) were conducted. Meta-regression analysis was used to evaluate the correlation between the cumulative dose of ATG and post-transplantation outcomes. Nonetheless, we did not assess the possible publication bias by funnel plots and Egger’s test since the number of studies included in each quantitative analysis was <10 (20). All analyses were performed using STATA SE 14.0 (StataCorp, College Station, TX, USA).

## Results

### Selection and Characteristics of the Included Studies

The initial literature search retrieved 531 relevant articles. Subsequently, eight RCTs that fulfilled the criteria were included in the final meta-analysis (10–12, 16, 17, 21–27). The search and selection process is illustrated in **Supplementary Figure 1**.

The characteristics of the included studies are summarized in **Table 1**. The eight studies encompassed 1,348 patients, including 683 cases in the ATG group and 665 cases in the control group. The median follow-up was 2 (range, 1–5) years. Five trials used rabbit ATG (Sanofi Genzyme, Cambridge, MA, USA) derived from human thymocytes, while three trials used rabbit ATLG (Neovii, Rapperswil, Switzerland) derived from the human Jurkat T cell line. Related donors were used in four studies, and the others used unrelated donors. The dose varied from 4.5 to 15 mg/kg for ATG and 10 to 60 mg/kg for ATLG. The overall methodological quality of included studies ranged from moderate to very low.

**Table 1:** Characteristics of studies included in the meta-analysis.

Study	Patients	Sample size		Age (year)		Gender, male (%)			Cumulative dose (mg/kg)	Regimen	Matching status, n (%)		
		ATG+ standard	Standard	ATG+ standard	Standard	ATG+ standard	Standard	ATG source			ATG+ standard	Standard	Follow-up
Bacigalupo et al., (21)	Unrelated sex match	29	25	28 (18–48)	29 (13–51)	NA	NA	Thymoglobulin	7.5	MAC	NA	NA	2 years
Bacigalupo et al., (21)	Unrelated sex match	27	28	32 (14–52)	28 (14–46)	NA	NA	Thymoglobulin	15	MAC	NA	NA	2 years
Bacigalupoet al., (23)	Related	84	86	NA	NA	NA	NA	Thymoglobulin	7.5	RIC/MAC	12 (14) 74 (88)	13 (15) 69 (80)	5 years
Finke et al., (25) Socie et al., (26) Finke et al., (24)	Related sex match	103	98	40 (18–60 )	39 (18–60)	56.3	59.2	ATLG	60	MAC	66 (64)	57 (58)	2 years, 3 years, 8 years
Soiffer et al., (11)	Unrelated HLA-matched	126	128	46 (18–64)	49 (19–65)	47.6	61.7	ATLG	60	MAC	NA	NA	2 years
Walker et al., (16) Walker et al., (27)	Unrelated sex match	99	97	49 (40–56)	49 (40–57)	64.00	67.00	Thymoglobulin	4.5	RIC/MAC	83 (84)	81(84)	1 years, 2 years
Bonifazi et al., (17) Kröger et al., (12)	Related sex match	83	72	39 (18–64)	43.5 (21–61)	64.00	56.00	ATLG	10	MAC	63 (76)	56 (78)	2 years, 5 years
Chang et al., (10)	Related HLA-matched	132	131	48 (40–61)	46 (40–58)	57.6	57.3	Thymoglobulin	4.5	RIC/MAC	132 (100)	131 (100)	3 years

NA, not applicable.

### GVHD

**Figure 1:**
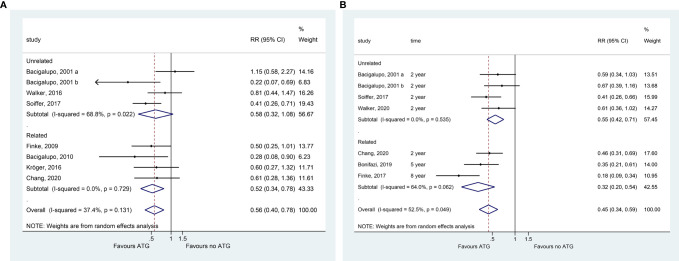
Forest plot for III–IV acute GVHD **(A)** and chronic GVHD **(B)**.

The overall pooled analysis of the eight studies showed that adding ATG to GVHD prophylaxis reduced the incidence of grade III–IV aGVHD (RR = 0.558, 95% CI = 0.400–0.779, P = 0.001; I^2^ = 37.4%; P_heterogeneity_ = 0.131; **Figure 1A**). The prevention of severe aGVHD was also observed in related (RR = 0.518, 95% CI = 0.343–0.782, P = 0.002), ATG (RR = 0.589, 95% CI = 0.378–0.917, P=0.019), and ATLG (RR = 0.457, 95% CI = 0.299–0.699, P < 0.001) subgroup analysis after prophylactic treatment with ATG. Moreover, ATG treatment significantly reduced cGVHD (RR = 0.446, 95% CI = 0.336–0.592, P < 0.001; I^2^ = 52.5%; P_heterogeneity_ = 0.049; **Figure 1B**) in all patients, and all subgroup analyses revealed similar benefits (**Table 2**). Meta-regression analysis showed that the cumulative dose of ATG did not affect both grade III–IV aGVHD and cGVHD (**Supplementary Figures 2A, B**).

**Table 2:** Summary of subgroup results.

Outcomes	N	RR (95%CI)	P	I-square, %	P(Heterogeneity)
III-IV aGVHD					
Overall	8	0.558(0.400,0.779)	0.001	37.4	0.131
Unrelated	4	0.584(0.316,1.077)	0.085	68.8	0.022
Related	4	0.518(0.343,0.782)	0.002	0	0.729
ATG	6	0.589(0.378,0.917)	0.019	44.8	0.107
ATLG	2	0.457(0.299,0.699)	<0.001	0	0.427
cGVHD					
Overall	7	0.446(0.336,0.592)	<0.001	52.5	0.049
Unrelated	4	0.549(0.424,0.710)	<0.001	0	0.535
Related	3	0.324(0.195,0.538)	<0.001	64	0.062
ATG	5	0.472(0.317,0.701)	<0.001	64.4	0.024
ATLG	2	0.386(0.273,0.546)	<0.001	0	0.634
OS					
Overall	8	0.922(0.736,1.155)	0.48	46.8	0.068
Unrelated	4	0.942(0.563,1.576)	0.821	75.6	0.006
Related	4	0.897(0.727,1.108)	0.314	0	0.9
ATG	6	0.848(0.708,1.016)	0.074	0	0.515
ATLG	2	1.219(0.577,2.574)	0.604	75.7	0.042
incidence of relapse					
Overall	8	1.201(0.970,1.488)	0.093	0	0.429
Unrelated	4	0.880(0.472,1.642)	0.688	52.6	0.097
Related	4	1.260(0.959,1.655)	0.097	0	0.947
ATG	6	1.064(0.815,1.390)	0.647	0	0.444
ATLG	2	1.495(1.045,2.140)	0.028	0	0.954
RFS					
Overall	4	1.008(0.627,1.622)	0.972	87	<0.001
Unrelated	1	1.319(1.054,1.651)	0.016		
Related	3	0.910(0.461,1.798)	0.787	88.4	<0.001
ATG	3	1.042(0.580,1.872)	0.89	91.2	<0.001
ATLG	1	0.890(0.514,1.542)	0.678		
NRM					
Overall	6	0.856(0.647,1.133)	0.277	18.3	0.295
Unrelated	2	1.004(0.415,2.432)	0.992	76.7	0.038
Related	4	0.769(0.563,1.050)	0.099	0	0.933
ATG	4	0.734(0.545,0.989)	0.042	0	0.872
ATLG	2	1.196(0.627,2.281)	0.588	41.9	0.189

### OS

**Figure 2:**
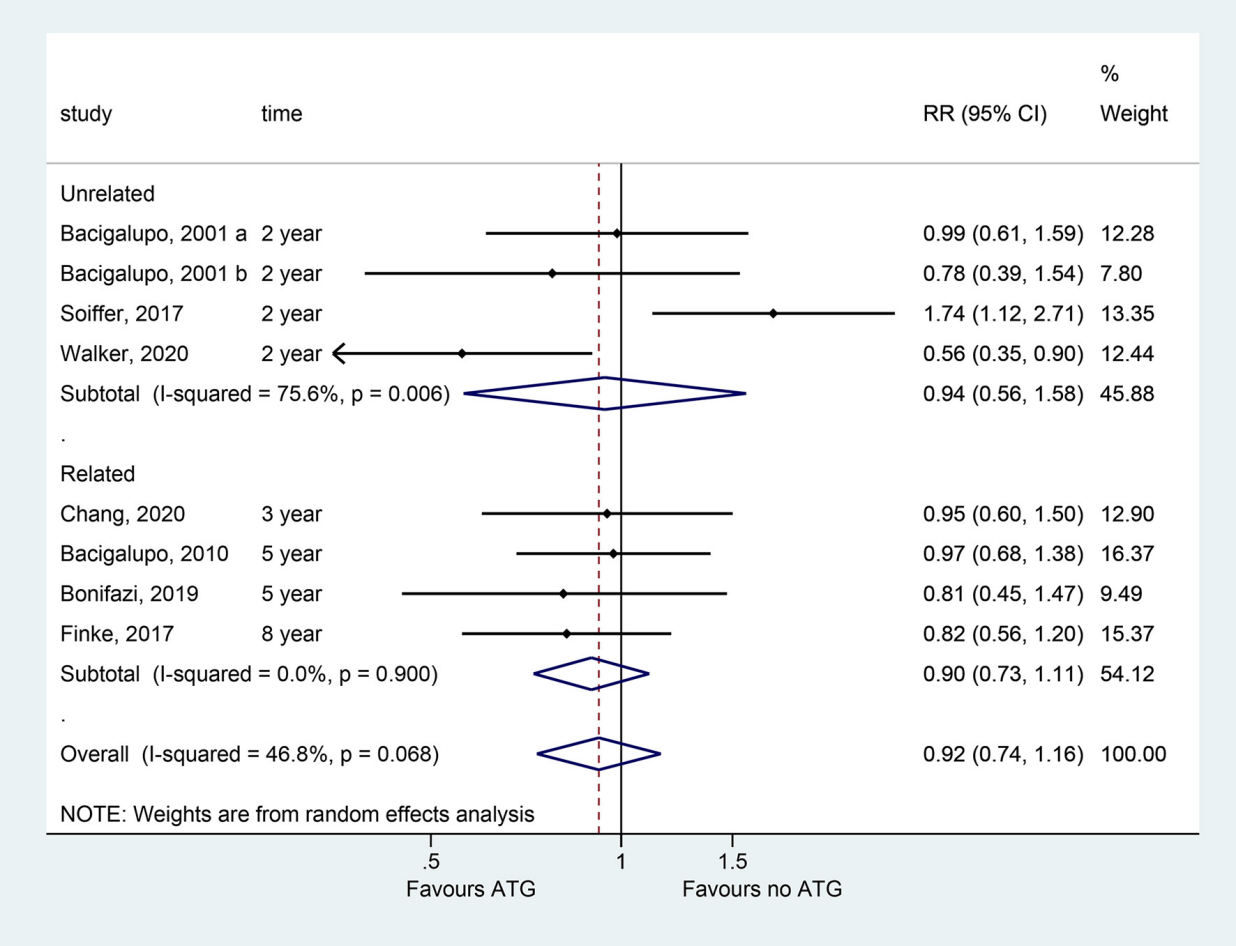
Forest plots of OS.

All eight studies presented OS data. Typically, patients treated with ATG did not benefit in terms of OS (RR = 0.922, 95% CI = 0.736, P = 0.48; I^2^ = 46.8%; P_heterogeneity_ = 0.068). In the subgroup analyses, no significant differences were detected in OS in the unrelated donors, related donors, ATG, and ATLG groups (**Figure 2** and **Table 2**). The meta-regression analysis showed that the cumulative dose of ATG did not affect the OS (**Supplementary Figure 2C**).

### Relapse

**Figure 3:**
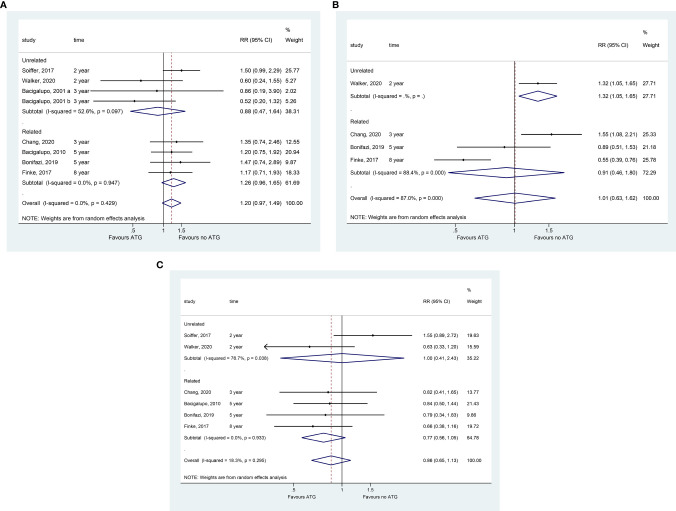
Forest plot for the incidence of relapse **(A)**, RFS **(B)**, and NRM **(C)**.

All eight studies presented relapse data. The incidence of relapse did not change with ATG in the whole sample (RR = 1.201, 95% CI = 0.970–1.488, P = 0.093; **Figure 3A**), as well as in the related, unrelated, and ATG subgroups. Adding ATLG in the conditioning regimen significantly reduced the risk of disease relapse according to the pooled analysis of four studies (RR = 1.495, 95% CI = 1.045–2.140, P = 0.028) (**Figure 3A** and **Table 2**). The meta-regression analysis showed that the cumulative dose of ATG did not affect relapse (**Supplementary Figure 2D**).

### RFS

Only one study suggested that ATG significantly reduced the RFS in patients undergoing transplantation from unrelated donors (RR = 1.319, 95% CI = 1.054–1.651, P = 0.016; **Figure 3B**). No significant decline was observed in RFS reduction in the overall and other subgroup pooled analyses (**Table 2**). The meta-regression analysis showed that the cumulative dose of ATG did not affect the OS (**Supplementary Figure 2E**).

### NRM

Adding ATG, but not ATLG, significantly improved NRM (RR = 0.734, 95% CI = 0.545–0.989, P = 0.042; **Figure 3C**). Compared to the no ATG group, ATG did not cause any significant difference in NRM based on the overall pooled analysis and related and unrelated subgroups analyses (**Table 2**).

### GRFS

**Figure 4:**
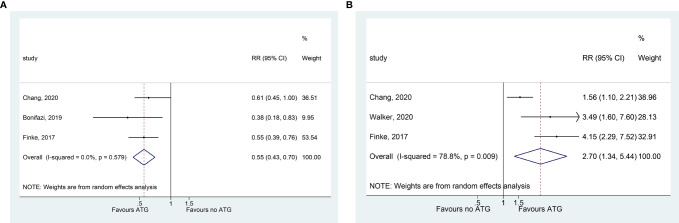
Forest plot for GRFS **(A)** and free of immunosuppressant therapy **(B)**.

Three studies assessing GRFS suggested that ATG with GVHD prophylaxis improved the GRFS (RR = 0.551, 95% CI = 0.432–0.703, P < 0.001) (**Figure 4A**).

### Free of Immunosuppressants

Compared with GVHD prophylaxis without ATG, a shorter immunosuppressive treatment was needed after the addition of ATG (RR = 2.700, 95% CI = 1.340–5.440, P = 0.005; three studies) (**Figure 4B**).

### Infection Complications

**Figure 5:**
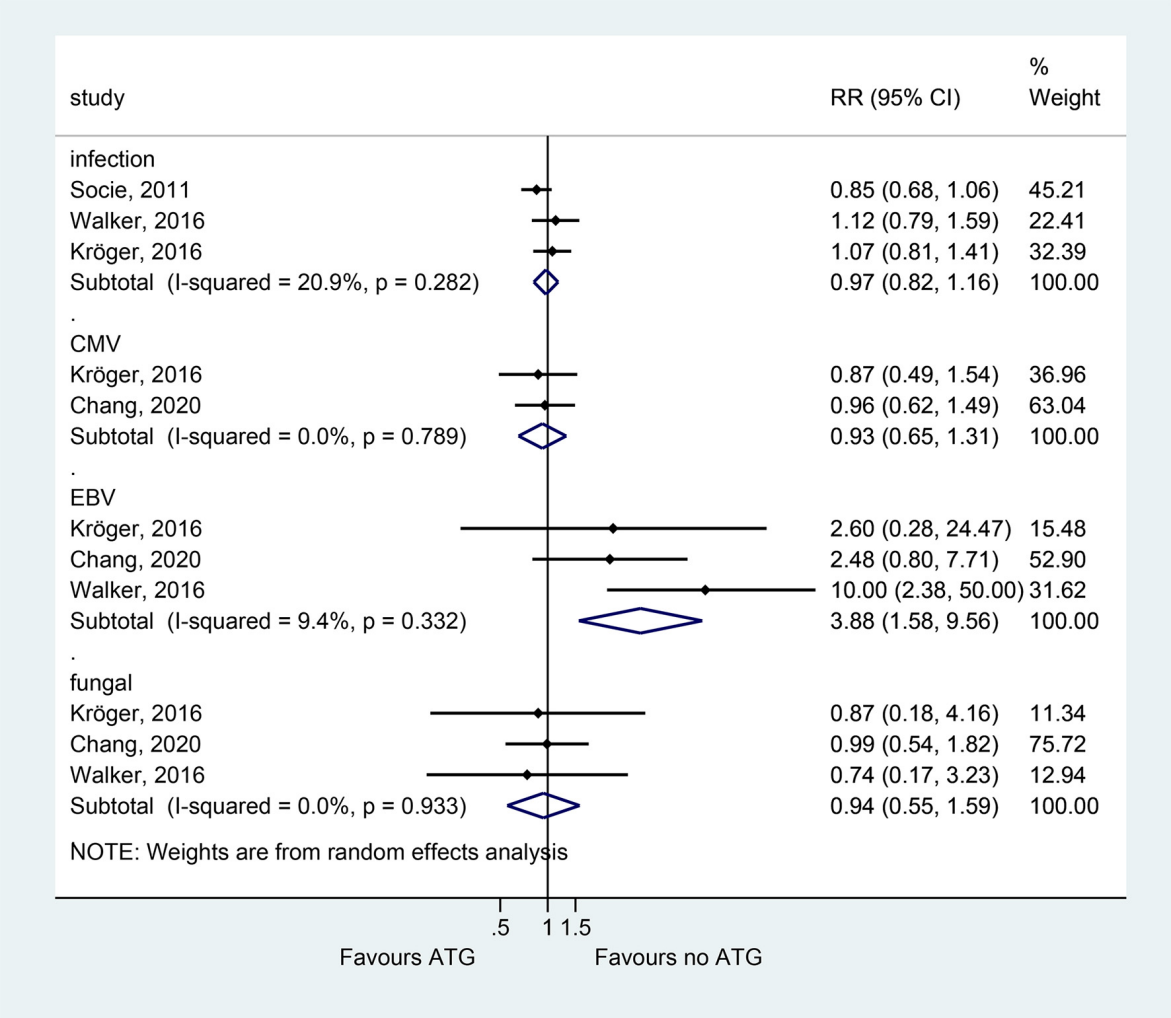
Forest plot for infection complications.

Epstein-Barr virus (EBV) reactivation (based on viremia monitoring) was common in patients who received ATG (RR = 3.882, 95% CI = 1.576–9.564, P = 0.003; three studies) (**Figure 4B**). However, GVHD prophylaxis with ATG did not significantly increase the total infection events, CMV reactivation, and fungal infections (**Figure 5** and **Table 2**).

## Discussion

This meta-analysis showed that the addition of ATG to GVHD prophylaxis in patients undergoing allo-HSCT resulted in a significantly lower risk of grade III–IV aGVHD and cGVHD and improved the GRFS of patients and subsequent freedom from immunosuppressants. Moreover, ATG treatment was correlated with a high incidence of EBV reactivation. No significant differences were noted regarding disease relapse and patients’ survival, including OS, RFS, and NRM.

In this study, some results were consistent with those of the meta-analysis by Kumar et al. (13), suggesting that ATG reduced grade II/III and grade III/IV aGVHD and cGVHD without affecting the OS and NRM. Still, the previous study showed an increased risk of relapse following the use of ATG, contradicting the present study results. The present updated meta-analysis included RCTs with extended follow-up, and further additional long-term survival benefits of better GRFS, improved OS, and decreased use of immunosuppressive therapy were revealed for GVHD prophylaxis with ATG (16, 17). Interestingly, a short immunosuppressive treatment regimen might reduce the hazards associated with long-term immunosuppression without increasing the disease relapse and infections. The significantly improved probability of survival without disease relapse and cGVHD might prove the long-term efficacy of the ATG plus standard conditioning regimen after allogeneic stem cell transplantation.

We also performed subgroup analyses according to the source of donors and the formulations of ATG to explore their impact on major transplantation outcomes. Although the overall analysis did not reveal any difference, subgroup analyses substantially altered some outcomes. The factors that could affect the transplantation outcomes included disease status at transplantation, different doses of ATG, and the types of donors and grafts; all those factors varied among the included studies. The pooled analyses of four RCTs suggested that ATLG plus GVHD prophylaxis significantly reduced the risk of disease relapse, whereas the addition of ATG significantly improved NRM based on one study. Thus, the type of ATG might be a related factor for relapse and NRM, but additional studies are necessary to confirm these results. In addition, RFS was significantly improved in patients undergoing transplantation from unrelated donors, suggesting that the type of donor also played a role in survival benefit. The overall analysis revealed that ATG treatment significantly decreased grade III/IV aGVHD. Still, the unrelated subgroup did not show a decreased risk of severe aGVHD. For the OS and cGVHD outcomes, the type of donor and ATG did not affect the overall conclusions.

The present study demonstrated that the reduced risk of severe aGVHD and aGVHD was not accompanied by improved relapse, OS, and NRM, which might be explained by other significant complications following prophylactic treatment. The systemic immunosuppressive conditioning regimens plus ATG might delay immune reconstitution and thus increase the risk of infectious complications. Thus, EBV was associated with the post-transplantation lymphoproliferative disorder (28, 29). Our pooled analysis showed that ATG increased subsequent EBV reactivation, while the incidence of general infection complications, CMV reactivation, and fungal infection did not change significantly. Previous reports had conflicting conclusions about whether ATG increased the incidence of infection. The meta-analysis by Yuan et al. found that the risk of infections was similar in the ATG and non-ATG groups and suggested that the anti-pathogenic immune defense was not completely compromised after inhibiting the T-cell pool (14).

Both dosage and timing of ATG have been shown to correlate with the outcomes after HSCT. We conducted a meta-regression analysis based on ATG cumulative dosage. The cumulative dose of ATG was not associated with post-HSCT OS, aGVHD, cGVHD, relapse, RFS, and NRM. Herein, we observed heterogeneity among the included studies. Therefore, subgroup analysis was carried out to identify the source, and the findings revealed that the heterogeneity in cGVHD was significantly decreased in the unrelated and ATLG subgroups, while for OS, the heterogeneity was significantly reduced in the related and ATG subgroups. Therefore, the ATG sources and patients’ correlation with donors could be deemed putative sources of heterogeneity.

The present meta-analysis also had limitations. The included studies were highly heterogeneous, which might be caused by different diseases, donor sources, formulations, and doses of drugs. Thus, meta-regression and subgroup analyses were performed to discuss the effect of heterogeneity on these results. In addition, only the RR could be extracted from some studies, not the HR, which might affect the accuracy of the results. Furthermore, the sample size of the included studies was relatively small, especially for the analysis of the outcome of GRFS and free of immunosuppressant therapy. Finally, the forest plots for GVHD, OS, and relapse took into consideration all eight papers, while all the other outcome parameters considered only one to six studies, limiting the conclusions. Thus, large-scale RCTs are required in the future to validate our findings.

In conclusion, adding ATG to GVHD prophylaxis prevents severe aGVHD and cGVHD, improves long-term survival endpoint GRFS, and reduces the use of immunosuppressive therapy without impact on disease relapse and patient survival. Moreover, ATG treatment increases the EBV reactivation, but it can be clinically non-significant. Thus, large sample studies are necessary to confirm these findings and to compare ATG and ATLG.

## Data Availability Statement

The original contributions presented in the study are included in the article/**Supplementary Material**. Further inquiries can be directed to the corresponding author.

## Author Contributions

XY carried out the studies, participated in collecting data, and drafted the manuscript. DL performed the statistical analysis and participated in its design. YX participated in the acquisition, analysis, or interpretation of data and drafted the manuscript. All authors contributed to the article and approved the submitted version.

## Funding

This study was supported by the Fund of Science and Technology of Sichuan Province (No. 2020YFS0253).

## Conflict of Interest

The authors declare that the research was conducted in the absence of any commercial or financial relationships that could be construed as a potential conflict of interest.

## Publisher’s Note

All claims expressed in this article are solely those of the authors and do not necessarily represent those of their affiliated organizations, or those of the publisher, the editors and the reviewers. Any product that may be evaluated in this article, or claim that may be made by its manufacturer, is not guaranteed or endorsed by the publisher.

## Supplementary Material

Supplementary Figure 1 Flow diagram of the study selection.

Supplementary Figure 2 Meta-regression plots for III–IV acute GVHD **(A)**, chronic GVHD **(B)**, OS **(C)**, incidence of relapse **(D)**, and NRM **(E)**.
