# Supplementary figures and images for: Anti-Thymocyte Globulin Prophylaxis in Patients With Hematological Malignancies Undergoing Allogeneic Hematopoietic Stem Cell Transplantation: An Updated Meta-Analysis
Source: Front Oncol. 2021 Aug 20;11:717678. doi: 10.3389/fonc.2021.717678 (PMC8417733; doi:10.3389/fonc.2021.717678)

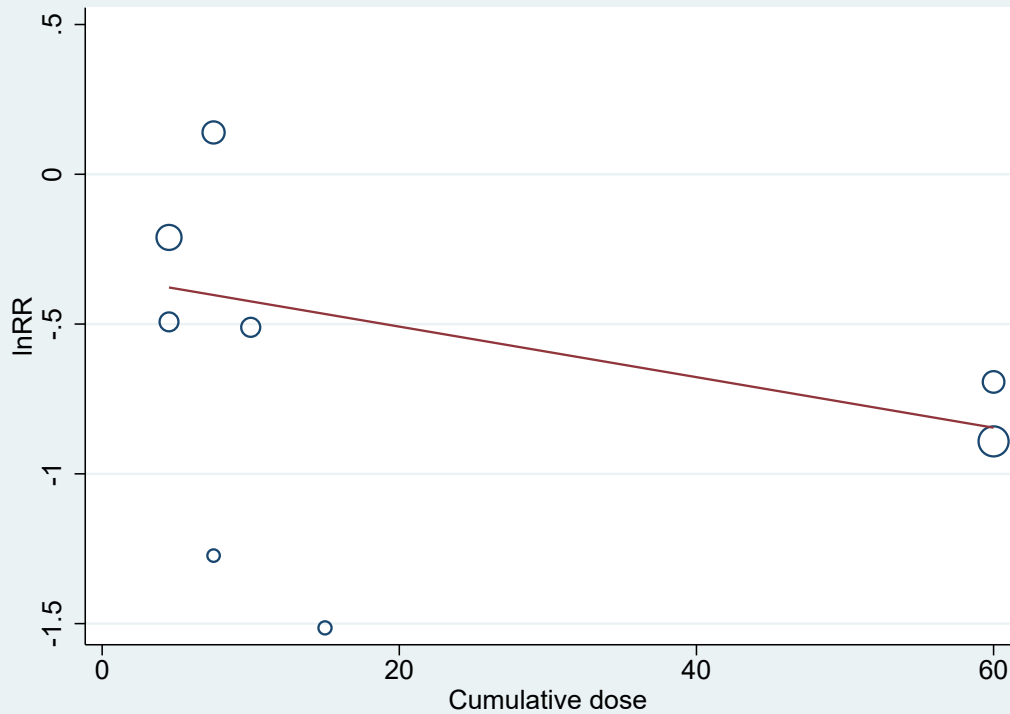

Supplement: Supplementary Figure 1 — Flow diagram of the study selection. [file DataSheet_1.pdf]

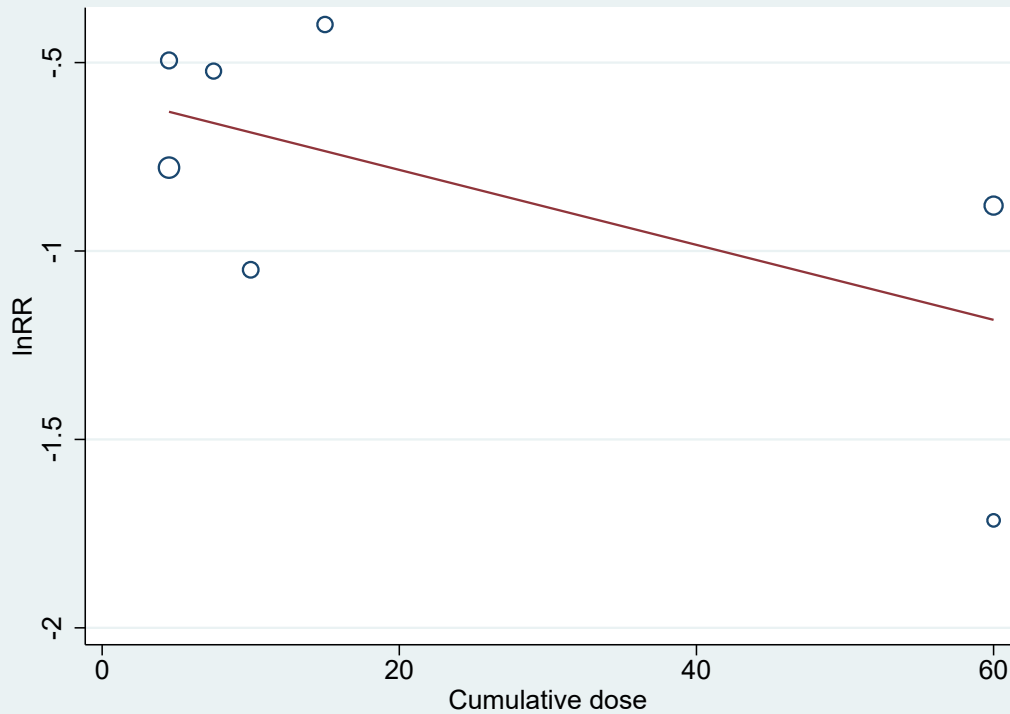

Supplement: Supplementary Figure 2 — Meta-regression plots for III–IV acute GVHD (A), chronic GVHD (B), OS (C), incidence of relapse (D), and NRM (E). [file DataSheet_2.pdf]

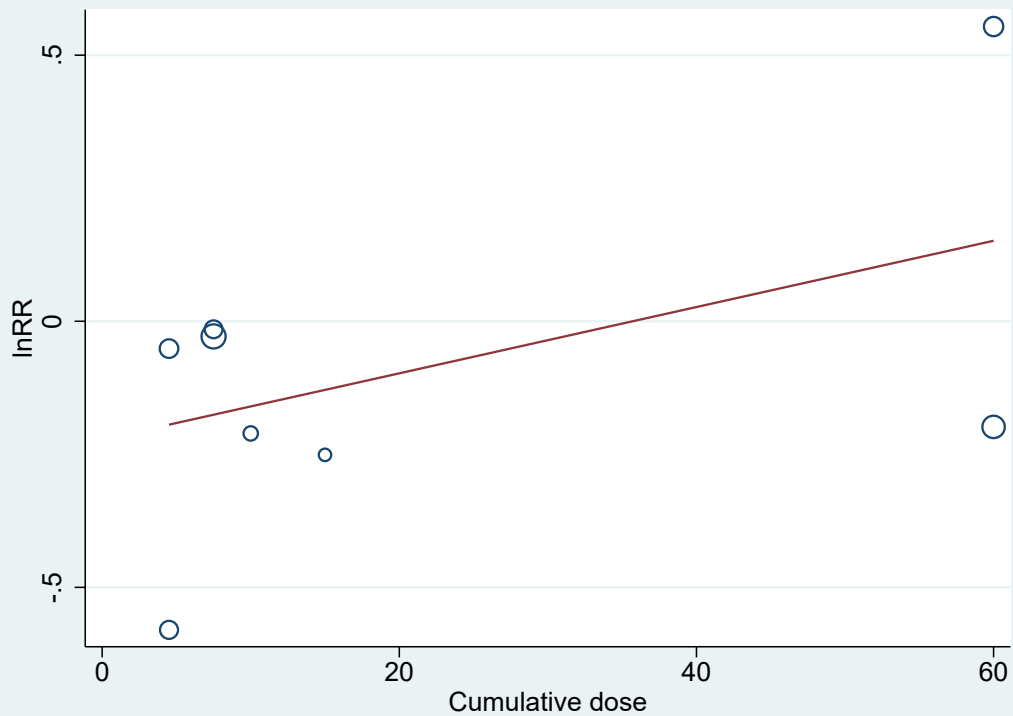

Supplement: Supplementary file 3 [file DataSheet_3.pdf]

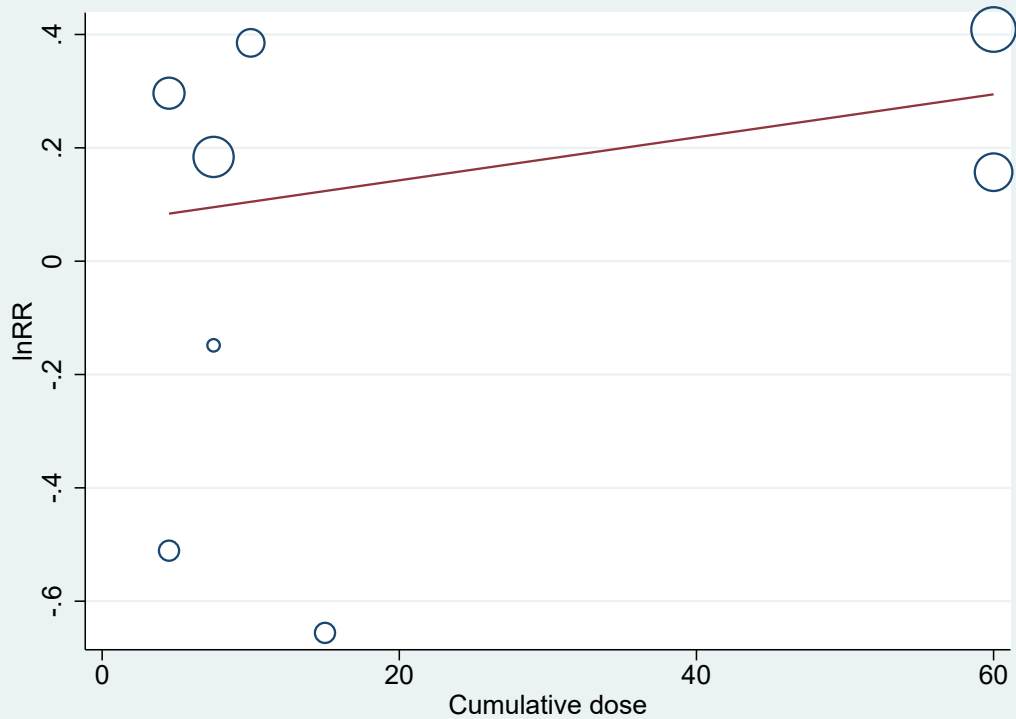

Supplement: Supplementary file 4 [file DataSheet_4.pdf]

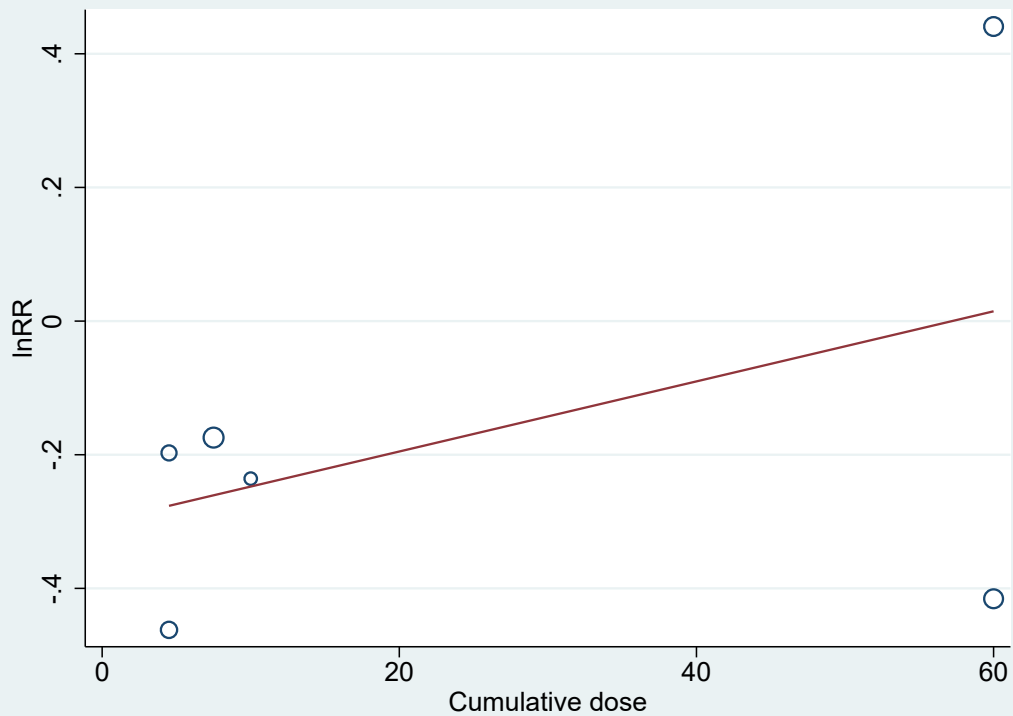

Supplement: Supplementary file 5 [file DataSheet_5.pdf]
