# Supplementary material for: Anti-Thymocyte Globulin Prophylaxis in Patients With Hematological Malignancies Undergoing Allogeneic Hematopoietic Stem Cell Transplantation: An Updated Meta-Analysis
Source: Front Oncol. 2021 Aug 20;11:717678. doi: 10.3389/fonc.2021.717678 (PMC8417733; doi:10.3389/fonc.2021.717678)
Supplement: Supplementary file 6 [file Image_1.docx]

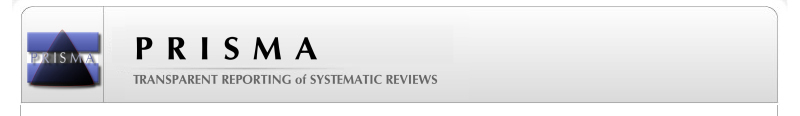
**PRISMA 2009 Flow Diagram**

Records after duplicates removed
(n = 112)

Records screened
(n =419)

## Identification

## Screening

Additional records identified through

manual searching
(n =2)

Records identified through database searching
(n =529)

PubMed (n=138);

Embase (n=258);

Cochrane (n=133)

Records excluded with reasons
(n =260)

Review (n=95);

Conference abstract (n=61);

Report/note (n=9);

Survey (n=2);

Letter (n=7);

Meta-analysis (n=12);

Editorial (n=5);

Expert opinion (n=4);

Guideline (n=1);

Others (n=38);

Full text could not be retrieved (n=26)

Full-text articles excluded, with reasons
(n =151)

Study aim/design (n=70);

Population (n=17);

Outcomes (n=12);

Intervention (n=52)

Full-text articles or abstracts assessed for eligibility
(n =159)

Studies included in qualitative synthesis

(meta-analysis)
(n = 8)

## Eligibility

## Included
