# Supplementary material for: Anti-Thymocyte Globulin Prophylaxis in Patients With Hematological Malignancies Undergoing Allogeneic Hematopoietic Stem Cell Transplantation: An Updated Meta-Analysis
Source: Front Oncol. 2021 Aug 20;11:717678. doi: 10.3389/fonc.2021.717678 (PMC8417733; doi:10.3389/fonc.2021.717678)
Supplement: Supplementary file 7 [file Table_1.docx]

| Study | Random sequence generation (selection bias) | Allocation concealment (selection bias) | Blinding of participants and personnel (performance bias) | Blinding of outcome assessment (detection bias) | Incomplete outcome data (attrition bias) | Selective reporting (reporting bias) | Other bias |
| --- | --- | --- | --- | --- | --- | --- | --- |
|  |  |  |  |  |  |  |  |
| Bacigalupo, 2001A | **Low** | **Low** | **Unclear** | **Unclear** | **Low** | **Unclear** | **Unclear** |
| Bacigalupo, 2001B | **Low** | **Low** | **Unclear** | **Unclear** | **Low** | **Unclear** | **Unclear** |
| Bacigalupo, 2010 | **Unclear** | **Unclear** | **High** | **High** | **Low** | **Low** | **Low** |
| Finke, 2017 | **Low** | **Low** | **Unclear** | **Low** | **Low** | **Unclear** | **Unclear** |
| Soiffer, 2017 | **Low** | **Low** | **Low** | **Unclear** | **High** | **Low** | **Low** |
| Walker, 2020 | **Low** | **Low** | **Unclear** | **Unclear** | **Low** | **Low** | **Low** |
| Bonifazi, 2019 | **Low** | **Low** | **Unclear** | **Unclear** | **Low** | **Low** | **Unclear** |
| Chang, 2020 | **Low** | **Unclear** | **Unclear** | **Unclear** | **Low** | **Low** | **Low** |

**Supplemental Table 1** Quality assessment of included articles
