# Supplementary material for: Anti-Thymocyte Globulin Prophylaxis in Patients With Hematological Malignancies Undergoing Allogeneic Hematopoietic Stem Cell Transplantation: An Updated Meta-Analysis
Source: Front Oncol. 2021 Aug 20;11:717678. doi: 10.3389/fonc.2021.717678 (PMC8417733; doi:10.3389/fonc.2021.717678)
Supplement: Supplementary file 8 [file Table_2.docx]

**Supplementary Table 2** Summary of subgroup results

| Outcomes | N | RR (95%CI) | P | I-square, % | P(Heterogeneity) |
| --- | --- | --- | --- | --- | --- |
| III-IV aGVHD |  |  |  |  |  |
| Overall | 8 | 0.558(0.400,0.779) | 0.001 | 37.4 | 0.131 |
| Unrelated | 4 | 0.584(0.316,1.077) | 0.085 | 68.8 | 0.022 |
| Related | 4 | 0.518(0.343,0.782) | 0.002 | 0 | 0.729 |
| ATG | 6 | 0.589(0.378,0.917) | 0.019 | 44.8 | 0.107 |
| ATLG | 2 | 0.457(0.299,0.699) | <0.001 | 0 | 0.427 |
| cGVHD |  |  |  |  |  |
| Overall | 7 | 0.446(0.336,0.592) | <0.001 | 52.5 | 0.049 |
| Unrelated | 4 | 0.549(0.424,0.710) | <0.001 | 0 | 0.535 |
| Related | 3 | 0.324(0.195,0.538) | <0.001 | 64 | 0.062 |
| ATG | 5 | 0.472(0.317,0.701) | <0.001 | 64.4 | 0.024 |
| ATLG | 2 | 0.386(0.273,0.546) | <0.001 | 0 | 0.634 |
| OS |  |  |  |  |  |
| Overall | 8 | 0.922(0.736,1.155) | 0.48 | 46.8 | 0.068 |
| Unrelated | 4 | 0.942(0.563,1.576) | 0.821 | 75.6 | 0.006 |
| Related | 4 | 0.897(0.727,1.108) | 0.314 | 0 | 0.9 |
| ATG | 6 | 0.848(0.708,1.016) | 0.074 | 0 | 0.515 |
| ATLG | 2 | 1.219(0.577,2.574) | 0.604 | 75.7 | 0.042 |
| incidence of relapse | |  |  |  |  |
| Overall | 8 | 1.201(0.970,1.488) | 0.093 | 0 | 0.429 |
| Unrelated | 4 | 0.880(0.472,1.642) | 0.688 | 52.6 | 0.097 |
| Related | 4 | 1.260(0.959,1.655) | 0.097 | 0 | 0.947 |
| ATG | 6 | 1.064(0.815,1.390) | 0.647 | 0 | 0.444 |
| ATLG | 2 | 1.495(1.045,2.140) | 0.028 | 0 | 0.954 |
| RFS |  |  |  |  |  |
| Overall | 4 | 1.008(0.627,1.622) | 0.972 | 87 | <0.001 |
| Unrelated | 1 | 1.319(1.054,1.651) | 0.016 |  |  |
| Related | 3 | 0.910(0.461,1.798) | 0.787 | 88.4 | <0.001 |
| ATG | 3 | 1.042(0.580,1.872) | 0.89 | 91.2 | <0.001 |
| ATLG | 1 | 0.890(0.514,1.542) | 0.678 |  |  |
| NRM |  |  |  |  |  |
| Overall | 6 | 0.856(0.647,1.133) | 0.277 | 18.3 | 0.295 |
| Unrelated | 2 | 1.004(0.415,2.432) | 0.992 | 76.7 | 0.038 |
| Related | 4 | 0.769(0.563,1.050) | 0.099 | 0 | 0.933 |
| ATG | 4 | 0.734(0.545,0.989) | 0.042 | 0 | 0.872 |
| ATLG | 2 | 1.196(0.627,2.281) | 0.588 | 41.9 | 0.189 |
